# Moderate benefit of escape room game on learning outcome in medicine

**DOI:** 10.1186/s12909-024-06352-8

**Published:** 2024-11-23

**Authors:** Peter Fedorcsak

**Affiliations:** 1 Institute of Clinical Medicine, Faculty of Medicine, University of Oslo, Oslo, Norway; 2 CRESCO Center for Embryology, Faculty of Medicine, University of Oslo, Oslo, Norway; 3 Department of Reproductive Medicine, Oslo University Hospital, Postbox 4950, Nydalen, Oslo, 0424 Norway

**Keywords:** Gamification, Escape room game, Learning outcome

## Abstract

**Background:**

Well-designed escape room games engage students with complex problems and challenge clinical and teamwork skills, but their impact on learning has been uncertain. This study aimed to estimate the effect size of escape room game on performance in a broad knowledge test.

**Methods:**

During clinical rotation in reproductive endocrinology and infertility (REI), medical students participated in a 3-hour small-group class. For 2 semesters, groups had traditional patient visits and case discussions, and for 3 semesters, patient visits and an escape room game including debrief. The game was set up in the outpatient clinic, the puzzles were taken from clinical problems in REI, and challenges included operating an ultrasound scanner on a mannequin. Mid-semester, students completed a test of general knowledge in REI. To estimate the effect of small group class on declarative knowledge, test scores of students who had already had the class (exposed) were compared to scores of those who had not yet had the class (control).

**Results:**

Students were highly satisfied with gamified teaching. Those who attended the small group class without the escape room game achieved similar scores on knowledge test than control students (Cohen’s *d* = 0.05, 95%CI -0.58 to 0.68, *n* = 71). Students who played the escape room game achieved marginally higher score than respective controls (Cohen’s *d* = 0.22, 95%CI -0.1 to 0.53, *n* = 182).

**Conclusions:**

Escape room game may improve learning outcome of a traditional small group class, but the effect of a single game session on declarative knowledge is modest and is unlikely to exceed related instructional methods like simulation.

**Supplementary Information:**

The online version contains supplementary material available at 10.1186/s12909-024-06352-8.

## Background

Clinical classes are often arranged in small groups, an instructional form that is effective for presenting and discussing case scenarios [1]. Although teachers and students prefer active involvement during clinical sessions [2], the classes may often fall back to the traditional grandstand model where students crowd around the clinician to observe the consultation [3]. Class arrangements promoting active participation like the supervising model or the report-back model [3], however, are seldom feasible in high-intensity or sensitive clinical settings, where one to one teaching may be appropriate [4], preferably combined with group-based activities that let learners to practice teamwork skills, discuss, present ideas, and persuade peers [1, 5]. Indeed, medical students self-identify as team workers [6], which may indicate a preference for learning situations requiring collective team effort, such as simulations and games.

Escape room is a live-action team-based game that stems from the point-and-click genre of adventure computer games, like the *Maniac Mansion*, *The Secret of the Monkey Island*, or the *Day of the Tentacle* (LucasArts). In an escape room, a team of players is locked in a physical room where they are presented a captivating story and a challenging task. By interacting with the environment under time pressure, discovering hidden clues and solving puzzles and riddles, the team must find a key to escape the room. The most successful game designs have been described by scholarly surveys [7]: Before the game, a mystical story is presented to the players to create anticipation and tension. Once in the room, most teams start with slow and careful discovery of the surroundings, but soon excitement will take over as the team members call out discoveries or hunch over puzzles in groups, and most will leave the game with excitement [7].

Nicholson [7] distinguishes four basic game formats depending on the presence of a specific theme and narrative, and whether the puzzles are stand-alone or integrated into the narrative. In the more elaborate games, the puzzles are part of the storytelling, and immersive experience comes from games designed with attention to player motivations, physical environment, intellectual challenges, and emotions [8].

The puzzle design is either path-based or sequential. In path-based designs, the team is presented with several different puzzles at the same time and each of the paths are needed to solve a meta-puzzle, which will unlock the next stage or victory [7]. The team can split into smaller groups to solve path-based puzzles. The sequential game design presents the players with one puzzle at a time that will unlock the next puzzle in sequence [7]. The sequential design requires the entire team work together. The most frequent types of puzzles are searching hidden objects; using light; counting; noticing something obvious in the room; symbol substitution with a key; using something in an unusual way; searching images; assembly of an object.

Escape room facilities usually provide a gamemaster to ensure fair player experience. The gamemaster may monitor the players via video, help the players if stuck or frustrated, and ensure safety [7]. Gamemasters may also give hints, either on request or at timed intervals. Nicholson’s survey [7] indicates that the average player’s success rate is 41%.

Escape rooms have become remarkably popular, with over 2200 event facilities established in the USA alone in 2020, where only a dozen existed in 2014 [9]. Educational use of escape rooms is also increasing [10], and specific tutorials are available for implementation in higher education [11], including undergraduate medical teaching [12, 13]. The escape room class designed by Friedrich et al. [12] aims to improve team skills of interprofessional health care students. After a plenary introduction, the students are divided into small groups (6–8) for the escape room experience. According to the storyline, the players must plan discharge from the hospital of a patient with diabetes and mental disease. The game by Friedrich et al. [12] is supported by a small collection of physical props (e.g. code-labelled balls hidden in the room), but most content is delivered to the players in online spreadsheets and text documents. The puzzles are elaborate but non-medical, including matching color to music tone, finding hidden objects, riddles, and an online maze, but the puzzles are not integrated in the game story. The escape room session is followed by a plenary debriefing to ascertain that the educational aims of the game had been met. The escape room game designed by Rosenkrantz et al. [13] aims to train collaboration and communication skills using a fictious game narrative (countering a zombie-like virus attack). Their game applies clinical props from emergency wards (ECG monitor, nebulizer mask), as well as toys (remote controlled car), and the riddles are aligned with the fictious narrative, i.e. triaging zombies [13].

Effective educational games deliberately capitalize on game features, including imaginary context, rules and goals, sensory aspects, level of challenge, complexity, and learner control, for educational aims [14]. According to the input-process-outcome game model, these features trigger a cycle of user judgment, reactions (enjoyment and interest), and behaviors (persistence), which result in self-motivated game play and achievement of training objectives [14]. The generic escape room game design is versatile and can be adapted to educational needs as shown by many examples [11–13], but efficacy of escape room in teaching and learning remains uncertain.

To improve educational outcomes of a traditional clinical class by exploiting the benefits of small group setting and games, I designed and implemented a serious escape room game for small group teaching in reproductive endocrinology and infertility (REI). Consecutive groups of students played the game during the semester, and the performance of students who played the game was compared to those who had not yet played the game on a mid-semester test of broad knowledge in REI, which allowed estimating the effect of escape room game on declarative knowledge. I also recorded students’ satisfaction with teaching and self-assessment of teamwork skills, to examine whether such factors were related to learning outcome.

## Methods

### Educational context and study design

The obligatory small group class in REI was part of the undergraduate curriculum for 5th year medical students at the Faculty of Medicine, University of Oslo. Permanent groups of 3–5 students were established at the start of the semester and were timetabled together for all teaching sessions. This was a non-randomized, retrospective, two group, posttest-only study assessing the effect of small group teaching or small group teaching combined with escape room as interventions during this class. Statistical power was not calculated in advance. Personal data of the students were not processed, and because of strictly anonymous design the need of ethical committee approval was waived, according to national regulations by Norwegian Agency for Shared Services in Education and Research.

### Small group class

The students were asked to prepare to the class and browse the REI website for texts, videos, podcast, and textbook chapters specifically relevant for the planned activities. The class started with clinical rounds, when each student shadowed a gynecologist for 2 h in the outpatient clinic. All students had had the opportunity to observe transvaginal ultrasound examinations, and most could also participate in consultations on early pregnancy. After the clinical rounds, the group was convened for either traditional case discussion (2 semesters) or escape room game and de-brief (3 semesters).

### Development of the game

Based on the generic escape room template [7], the game was developed with the principles of educational games in mind, emphasizing integration of the learning objectives and the game objective [15]. The learning objectives were:

1. Describe the process of spontaneous human conception, assisted reproduction treatment, the signs of intrauterine pregnancy (IUP) and extrauterine gestation.
2. Review clinical records and perform ultrasound examination of the pelvic organs on the mannequin, including assessment of the fetal crown-rump length (CRL).
3. Recognize clinical, biochemical, and ultrasonographic signs of early IUP and calculate the gestational age using multiple cues.
4. Demonstrate professional attitude during clinical examination.
5. Demonstrate efficient teamwork under time pressure.

Based on the learning objectives, practical clinically relevant puzzles were drafted and integrated into a game story. The draft game was tested in trial sessions to refine the storyline, rules, physical aspects, level of challenge, and complexity with learning goals and remove distracting elements [14].

### Implementation of the escape room game

**Fig. 1:**
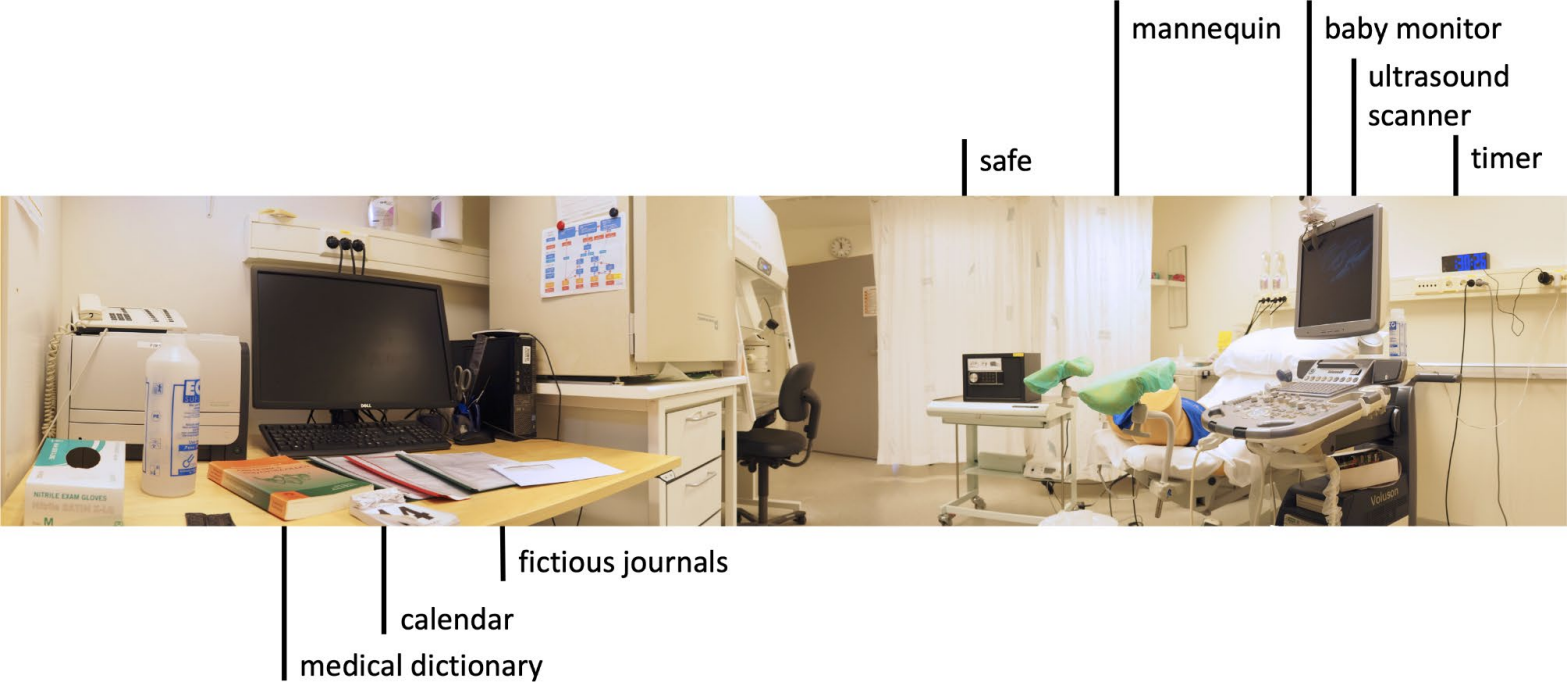
Arrangement of game items in the outpatient room. Before starting the escape room game, the gamemaster arranges all props and clinical items in the room (left to right): the medical dictionary, tear-off calendar showing the game’s date and fictious journals on the desk; document safe with the gestational wheel and address book locked inside; mannequin on the examination chair; ultrasound scanner; baby monitor to view the examination area; large-display countdown timer set to 30 min

Detailed instructions for setting up the game, including sourcing of props, gamemaster’s script, and fictious patient journals is given in Supplemental online material. Briefly, the game was set up in a gynecological outpatient room. Gynecological examination chair, ultrasound scanner with transvaginal transducer, and gestational wheel were borrowed from the clinic. Props specifically purchased for the game included a medical dictionary, intrauterine pregnancy transvaginal ultrasound training mannequin, a document safe with digital lock, countdown timer with large LED display, and a baby monitor with camera. Additional items were an envelope with the welcome letter and the safe code puzzle, a tear-off calendar, three fictious patient journals, and an address book with the telephone number puzzle. The gestational wheel and the address book were locked in the document safe, the other items were inconspicuously arranged in the room (Fig. 1).

After arranging the props in the room, the gamemaster came out to the corridor to greet the waiting students, read out the introductory script, gave the students the envelope with the game quest, and let the group enter the room. The group was also informed that the gameplay will be observed on the baby monitor but no recordings will be made. The countdown timer in the room was set to 30 min.

The objective of the players was to examine the mannequin using the ultrasound scanner, select the patient journal that matched ultrasound findings on the mannequin, unlock the safe, decode the telephone number in the address book, and call the number to tell the fictious patient about follow-up investigations. Groups making the phone call within 30 min and giving appropriate instructions won the game.

**Fig. 2:**
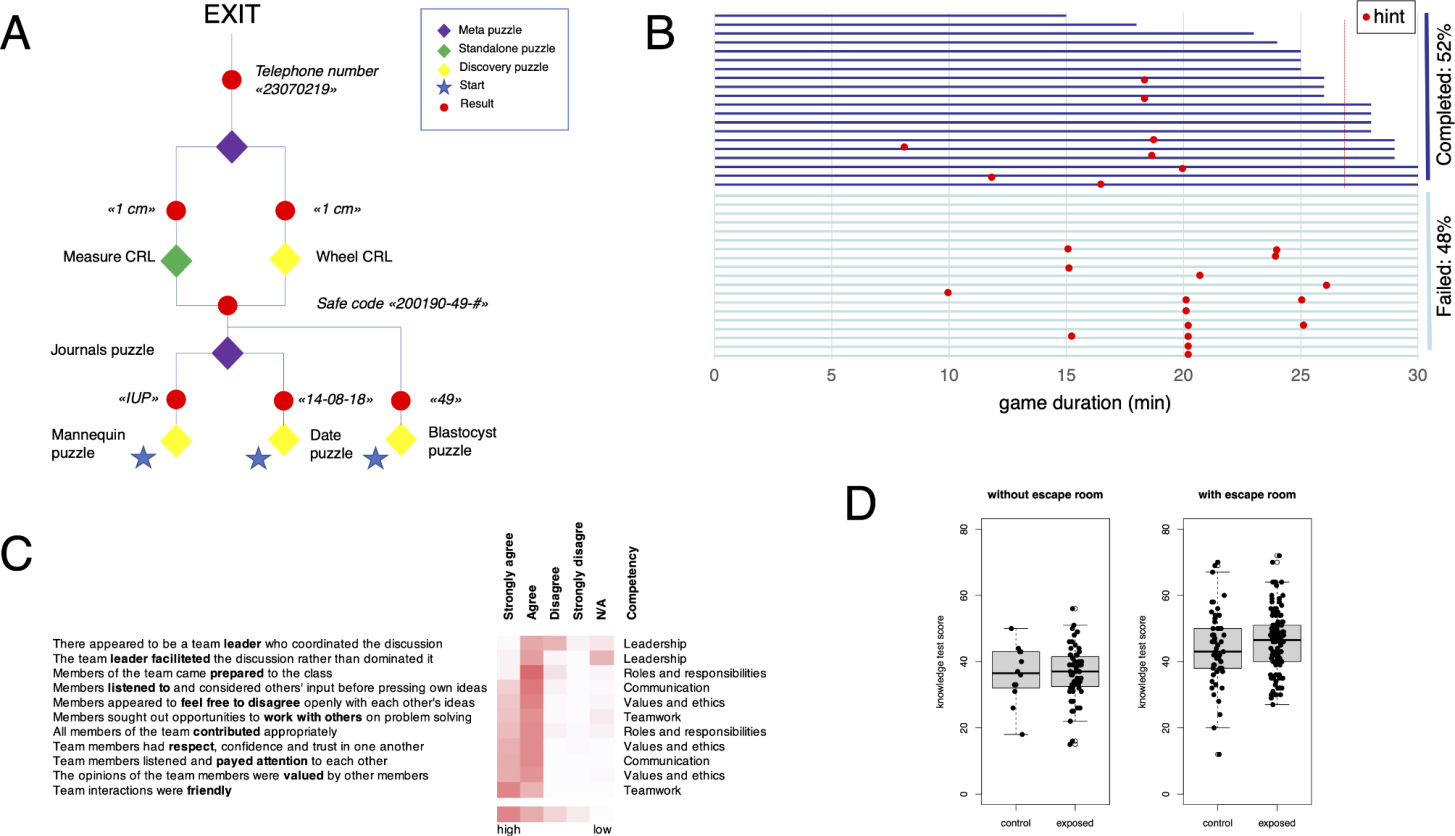
Schematic chart of the puzzles, students’ performance during the game and test of knowledge. *A*, the game comprised 6 puzzles that all needed to be solved for successfully exiting the game. For discovery puzzles, players needed to locate objects, e.g. find the calendar for the date puzzle. Meta-puzzles used solution of one or more other puzzles. *B*, progress chart of 39 gameplays with hints indicated as red dots. *C*, students self-rated their teamwork using 11 items from the Jefferson Teamwork Observation Guide [16]. Average frequency of responses from 56 students is illustrated with a color density scale. *D*, distribution of knowledge test score in students after small group teaching with or without escape room game (exposed) versus students who had not had small group teaching (control)

The game contained 8 interconnected puzzles of varying complexity and difficulty (Fig. 2A). The date puzzle required finding the desktop calendar and recognizing the fictious date of the game. The blastocyst puzzle required finding the page number for entry ‘blastocyst’ in the medical dictionary by identifying drawing of a blastocyst. To solve the IUP puzzle, the group needed to operate the ultrasound scanner, examine the mannequin, and identify 8 weeks old intrauterine pregnancy. The journals puzzle required solution of the calendar puzzle, as well as correct interpretation of three fictious patient histories, which were extrauterine pregnancy, first trimester pregnancy conceived by IVF, and second trimester pregnancy conceived spontaneously. The journals puzzle and the blastocyst puzzle unlocked the safe. When opening the safe, the group needed to solve the CRL puzzle, which required either measuring the approximate fetal length on the mannequin or use the gestational wheel found in the safe. Solution of the CRL puzzle gave the missing digit in the telephone number, allowing the group to make the call. (The phone number belonged to the gamemaster.) The correct instruction to the patient was that she was pregnant and advised to book appointment for prenatal visits week 12 of gestation at her GP, as it is recommended in Norway. Unless the group finished earlier, the game was ended after 30 min on the chime of the countdown timer.

The gamemaster followed the group through the baby monitor and noted progress, misunderstandings, errors, and other discussion points for the debriefing. Hints were given through the baby monitor if requested or if the group was stuck in the game. The game was always followed by 30–45 min structured debriefing.

### Student responses

The students were invited to answer a 14-item questionnaire on the satisfaction with the class, teamwork quality using the Jefferson Teamwork Observation Guide [16], new knowledge learned, and suggestions for improvement. A secure anonymous webform service from the University of Oslo was used. The student evaluation form is available as supplementary material. Quantitative questionnaire data were evaluated using descriptive statistics. Open text responses were summarized by the reflexive thematic analysis method of Braun and Clarke [17]. Because of the narrow focus of questions and brevity of responses (mean word count 40, range 2–173), an inductive-realist approach was followed. Initial exploration and iterative refinement resulted in a set of codes that were used to mark text segments: anatomy, physiology, practice of clinical skills, theory of clinical examination, therapy (theme new knowledge learned); communication, leadership, relational (theme teamwork aspects); escape room, organisation and planning, web material, hands-on practice (theme positive or negative aspects of teaching). A single coder evaluated responses and intercoder reliability was not calculated [18].

### Test of knowledge

All students participated in one non-obligatory plenary session on REI during the semester. As part of this session, students filled out an individual test of 20 multiple choice questions. The questions addressed broad knowledge in REI and were not targeted to specific topics of the small group class and the escape room game. As the plenary session was held mid-semester, some students had already had the small group class in REI by the time of the session while others had not. To estimate the effect of small group class on declarative knowledge, test scores of students who had already had the class (exposed) were compared to scores of those who had not yet had the class (control). The effect size was expressed as Cohen’s *d*.

## Results

During 5 semesters in 2017 to 2019, small groups of 2–5 students attended the outpatient class in reproductive endocrinology and infertility (REI). The class started with patient visits followed by either traditional case discussion for 2 semesters (56 groups, 213 students) or the escape room game for 3 semesters (84 groups, 324 students). Most groups (51%) completed the game within median 27 min (range 15–30 min) after receiving one or more hints, usually after 17 min into the game (Fig. 2B).

### Student evaluation

Students rated satisfaction with the teaching, teamwork quality, and learning outcome. During the 2 semesters without escape room game, 36 (54%) of 67 students (31% response rate) reported high overall satisfaction with teaching (grade 1 on scale 1–5). During the 3 semesters with escape room game, 63 (66%) of 95 respondents (29% response rate) expressed high overall satisfaction with the class. Mean student satisfaction during the two periods was comparable (1.41 and 1.49 for the respective periods; mean difference = 0.08, 95%CI -0.01 to 0.18).

Short open text responses from 95 students participating in the escape room game included 163 positive, 166 neutral, and 68 negative comments. Students most often commended the escape room (*n* = 63) and organization of the class (*n* = 57), whereas the topic of critical comments was also organization (*n* = 46) and the escape room (*n* = 10).

The students were also asked to self-evaluate the quality of teamwork during the escape room game. The respondents (*n* = 56) characterized the teamwork as friendly and respectful, but lacking leadership (Fig. 2C). Friendliness without leadership was a recurring theme of the open text responses:

*« A bit chaotic because there was no teamleader. »*

*«It’s nice to be able to discuss findings and ideas with one another and together find the right answer.»*.

*«Although I wasn’t sure about everything*,* I trusted my team-mates and together we got the solution of the game.»*.

*«It is possible to come to the right conclusion*,* and then in plenary discuss ourselves away from it*,* never to end up in the right place again.»*.

The students most often reported learning new clinical skills (*n* = 20), like using the gestational wheel, operating the ultrasound scanner, and measuring CRL, new knowledge about treatment (*n* = 9), like IVF, as well as new knowledge in physiology (*n* = 8), like gonadotropin action or early pregnancy physiology (Table 1).

**Table 1:** Distribution of self-reported learning outcome (*n* = 49) by 95 students participating in a clinical class including the escape room game

Learning outcome	Selected responses
Practiced clinical skills (*n* = 20; 41%)	*“A taste of how ultrasound works.”* *“To properly use the [pregnancy] wheel.”*
Knowledge on treatment (*n* = 9; 18%)	*“What missed abortion is*,* and what we should measure when we do an ultrasound scan.”* *“Details of IVF treatment.”*
Knowledge on physiology (*n* = 8; 16%)	*“Learned that we have to stimulate the follicles with FSH.”*
Anatomy (*n* = 6; 12%)	*“Learned about the fornices of the vagina and more about orientation on the screen.”* *“Location and significance of the cul de sac.”* *“Anteverted vs retroverted uterus.”*
Theory of clinical examination (*n* = 6; 12%)	*“How to use the ultrasound probe to get an appropriate picture of the uterus and adnexa.”*

### Test of knowledge

Students who attended the small group class without the escape room game achieved similar scores on broad knowledge test in REI than control students (Cohen’s *d* = 0.05, 95%CI -0.58 to 0.68, *n* = 71). Students who played the escape room game achieved marginally higher score than respective controls (Cohen’s *d* = 0.22, 95%CI -0.1 to 0.53, *n* = 182, Fig. 2D).

## Discussion

This paper reports design and implementation of a serious escape room game in clinical small group teaching. The game is moderately difficult for undergraduate medical students, while it gives a satisfactory experience and invokes persistent task-focused team effort. Feedback from students suggests that the game was able to engage with intended learning content, including practicing examination and diagnostic skills, and retention of subject-specific knowledge.

The effect of gameplay on acquisition and recall of broad declarative knowledge was moderate (effect size 0.22, 95%CI -0.1 to 0.53) compared to related teaching methods. Indeed, a meta-analysis found large effect of educational games on cognitive (0.49), motivational (0.36), and behavioral (0.25) outcomes [19], further exceeded by the effect of technology-enhanced simulation on knowledge (1.20), skills (1.09 to 1.18), behavior (0.79), and patient care (0.50) [20]. More broadly, active learning interventions may achieve higher average improvement in student attainments (effect size 0.47), particularly in smaller class sizes [21], than seen in this study. However, several factors may contribute to the moderate observed effect size: (1) The MCQ test assessed broad declarative knowledge in REI, not limiting the questions to specific topics encountered during the small group class, and the effect size on more focused learning may thus be underestimated. (2) The study did not assess other relevant learning outcomes, including skills, behaviors or patient care. (3) Educational games may promote learning by increasing engagement, motivation and awareness of a subject [14], which were not measured by the MCQ test. (4) A single 1 h session may be too short to trigger repeated game cycles and reinforce learning [14]. Indeed, in the context of simulation, extending trainings over 1 day were shown to be associated with better learning outcomes [20]. (5) Nonetheless, given the present sample size, the wide confidence interval (95%CI -0.1 to 0.53) may be consistent with higher true mean effect size than observed.

### Learning process

Serious games promote learning by allowing repeated active engagement with the game environment coupled with appropriate instructional support, including learning content, scaffolding and debriefing [14]. This game allowed free roaming in the gamespace and interaction with riddles, medical instruments and game props, each with specific educational purpose. The learning content was scaffolded multiple ways, including website and videos for advance viewing, and one-to-one clinical teaching. Discussion during the de-briefing session, which is thought to provide a link between the game experience and real-world educational outcome [14], usually touched on the group’s game experience, solution strategies, mistakes and errors, which were sometimes only uncovered by watching the gameplay with baby monitor. To reinforce the educational aims of the class, the debriefing often ended by demonstration of proper examination technique on the mannequin, pelvic ultrasound anatomy, early pregnancy signs, calculation of gestational age, and interpretation of the menstrual calendar. Self-evaluation by the students a few days after class suggests that some learned facts were retained.

Emotions are powerful drivers of motivation and sustain engagement in games, and therefore creating player enjoyment is seen as the key objective of gamification [22]. High satisfaction reported by the students and positive emotions as excitement, amusement, and surprise that I have observed as gamemaster were probably contributing to improved learning outcomes. Students also reported emotionally positive experiences during teamwork, which was characterized by friendliness, respect and attention. The learning processes during this escape room game remain nonetheless to be understood. Notably, the escape room game setting and camera observation may allow studying student-to-student communication processes, which have important applications for team-based instruction [23].

### Subjective observations

In my experience, it is useful during class to wait revealing the game right until it starts, in order to exploit surprise and maximize initial excitement. During the game students showed sustained intense involvement, and I have usually seen persisting task-focused effort. The groups worked even more ferociously towards the end, which they were constantly reminded by the countdown timer. Nonetheless, some groups found the riddles overtly challenging. Being able to give timely hints through baby monitor may have reduced frustration, improved game experience, and kept motivation sustained.

I observed various problem-solving strategies, like reliance on shortcut cues, but seldom trial-and-error approach. Students showed professional conduct while alone in the room, for example all followed used gloves and transducer sheath.

In agreement with students’ self-reports, I often observed inefficient teamwork, lack of self-management, and non-emergence of leader, which were sources of frustration and inefficiency. Indeed, some groups struggled to recall from transactive memory even the most recent knowledge, for example diagnosis of intrauterine pregnancy, even though they had just participated in early pregnancy consultations and could watch training videos before class. I have only occasionally noted emergence of a leader who assigned tasks, summarized status, or prevented the group going off the track.

Location, props, and puzzles were selected for authentic experience and to increase the players’ identification with the storyline. Pilot versions of the game were vetted for clinical relevance, and distracting items, riddles, or story elements were removed from the final game. Indeed, meta-analysis of serious games suggests that distractive narratives can reduce learning effect [24]. To the extent it aspired to represent reality, this escape room was more a simulation than a game [14]. The gameplay required a broad set of knowledge and skills, including basic physiology and clinical examination, and could not be solved by a single player within the allocated time, thus forcing teamwork. Riddles and activities also challenged boldness and resolution of the group, for example by needing to operate an unfamiliar ultrasound scanner for the first time.

### Practical aspects of implementation

Requirements of time, space, costs, and group size may hinder implementation of escape room games. The gamemaster must allocate 10–15 min ahead of the activity to find a vacant room and arrange the props while keeping eye on student safety. Further 30 min are needed for the gameplay, 30–45 min for debriefing, and 5–10 min for packing down. A suitable clinical room with ultrasound scanner should be allocated, which can be challenging in a busy clinical environment. The props, especially the mannequin, are expensive. The game can be optimally played by a group of 3–5 medical students, which may limit its versatility. In my experience, two students could be overwhelmed by the workload, while some students in a group of 6 or more will idle. One gamemaster would be unable to monitor more than two games at the same time, and it may be impractical to set up simultaneous games because of multiplication of costs. For large groups, I created a boxed version of the game replacing the ultrasound scanner and mannequin with a pre-recorded video. The boxed version can be played concurrently by many groups of students (tested with 24 groups), but the valuable player experiences of a real clinical environment are lost.

### Limitations

The non-randomized posttest-only design of this study cannot rule out that factors related to learners or teaching during the compared periods, rather than the escape room activity, explain the observed effects on learning. Furthermore, the specific settings of the game, including the stage in medical education, the age of learners, and the subject taught, may not be transferrable to other educational situations. Nonetheless, successful implementation of escape rooms in many educational settings [11–13] suggests that the generic game template used here was versatile. Escape room activities are currently very popular among young adults, but whether this innovation will be adopted beyond the hype sequence is uncertain [25].

## Conclusions

In summary, escape room is an engaging and popular activity that can be implemented in small group clinical teaching. The concept is flexible, allows many iterations, and can be easily modified or adjusted for the target learners. For clinical teaching, it may be appropriate to design games with relevant narrative and non-distracting clinical puzzles. However, escape rooms are time-consuming to create and supervise, and optimal experience may require expensive props and resources. The effect of escape room games on learning outcomes may be narrow and moderate.

## Electronic supplementary material

Below is the link to the electronic supplementary material.

Supplementary Material 1

Supplementary Material 2

## Data Availability

Supplemental online material gives comprehensive description of the escape room game allowing replication and free adaptation.

## Declarations

### Clinical trial number

This study does not report on a health care intervention and was not registered as a clinical trial.
