# Supplementary material for: Moderate benefit of escape room game on learning outcome in medicine
Source: BMC Med Educ. 2024 Nov 23;24:1353. doi: 10.1186/s12909-024-06352-8 (PMC11585938; doi:10.1186/s12909-024-06352-8)

UiO : Institute of Clinical Medicine
University of Oslo

Guidebook for serious escape room game in gynecology

Peter Fedorcsak MD PhD
professor

14-JULY-2024

Table of contents

Introduction 2
Educational objectives 2
  Relevant APGO curricular objectives 2
Background 3
  Serious games 3
  Escape rooms 3
  Curricular context 4
  Prerequisite knowledge 4
  Educational context 4
Implementation 5
  1. Safety 5
  2. Time requirements 5
  3. Group size 5
  4. Location 5
  5. Equipment, props 6
  6. Preparations 6
  7. Starting the game 7
  8. Gameplay 8
  9. Ending the game 8
  10. De-briefing 8
  11. Post-class activities 8
  11. Evaluation 9
References 10
Appendix 11
  A. Flowchart 11
  B. Description of the puzzles 12
  C. Letter from the doctor 14
  D. Journals 15
  E. Address book page 20

Introduction

This guidebook describes implementation of escape room game in reproductive endocrinology and infertility (REI). The game gives hands-on simulated clinical experience to students and integrates well into the outpatient clinical teaching in REI.

Educational objectives

By the end of this activity, learners will be able to:
1. Describe the timings of spontaneous conception and assisted reproduction treatment, the signs of intrauterine pregnancy and ectopic gestation.
2. Review clinical records and perform ultrasound examination of the pelvic organs on the mannequin, including assessment of the fetal crown-lump length.
3. Recognize clinical, biochemical, and ultrasonographic signs of early intrauterine pregnancy and calculate the gestational age using multiple cues.
4. Demonstrate professional attitude during clinical examination.
5. Demonstrate efficient teamwork under time pressure.

Relevant APGO curricular objectives

4. Diagnosis and management plan
   A. Generate a problem list
   B. Formulate diagnostic impression, including differential diagnosis
   C. Develop a management plan
5. Personal interaction and communication skills
   B. Work cooperatively
   C. Analyze his/her own strengths in interaction and communication skills
8. Maternal-fetal physiology
   C. Interpret common diagnostic studies during pregnancy
10. Antepartum care
   A. Diagnose pregnancy
   B. Determine gestational age
   C. Describe appropriate diagnostic studies and their timing for normal pregnancy
15. Ectopic pregnancy
   A. Develop a differential diagnosis for vaginal bleeding and abdominal pain in the first trimester
   B. List risk factors for ectopic pregnancy
   D. Discuss diagnostic protocols for ectopic pregnancy
   E. Describe treatment options for patients with ectopic pregnancy
48. Infertility
   A. Define infertility
   B. List the causes of infertility
   C. Describe the evaluation and management of an infertile couple
   E. Describe management options for infertility

Background

Serious games

Gamification is a generic term to describe application of game design principles in non-game contexts [1]. These principles are game mechanics, dynamics, and emotions, also called the MDE-framework. Game mechanics refer to the pre-defined rules, settings, context, and goals of the game, which remain constant for all players. Dynamics are the type of player behavior that emerge during the game, including cooperation, competition, or cheating. Emotions are the affections in the player when participating in the gamified experience, such as excitement, surprise, or disappointment. A gamified experience designed along these principles will touch the drivers of human behavior, such as reinforcement, rewards, and emotions, and may thus change the behavior of the player and bring about a desired outcome.

As games are increasingly recognized tools in education, gamified learning experiences become more and more implemented in teaching. Indeed, there are many examples of serious games in medical education, with some evidence of success. The point-and-click adventure game *Uro-Island* teaches urine analysis and was found to improve students' knowledge and learning experience in a randomized study [2]. A puzzle game of cardiac physiology reduced exam mistakes and improved students' perception of learning compared to traditional "full" lecture [3]. Implementation of games in small group classes of pharmacology was associated with positive student experience and improved participation [4].

A recent systematic review identified 21 publications that described the pedagogic strategies and the educational value of computer games in medical education [5]. Sixteen of 21 games (76%) Gorbanev et al. reviewed, applied either quiz or simulation to convey facts and develop skills through repetition, and only one game, the *Uro-Island*, created an adventure scenario. The reviewed papers most often documented improvement in knowledge and skills and none assessed changes in learners' behavior or patient outcome [5]. The specific game design elements that promote medical learning remain to be identified [6].

Escape rooms

Escape room, or room escape, is a live-action team-based game that stems from the point-and-click genre of adventure computer games, like the *Maniac Mansion*, *The Secret of the Monkey Island*, or the *Day of the Tentacle* (LucasArts). In an escape room, a team of players is locked in a physical room where they are presented to a captivating story and a challenging task. By interacting with the environment, discovering hidden clues and solving puzzles and riddles, the team must find a key to escape the room. Time pressure is a core element of the game.

The most successful game designs have been described by scholarly surveys [7]:

Before the game, a mystical story is presented to the players to create anticipation and tension. Once in the room, most teams start with slow and careful discovery of the surroundings, but soon excitement will take over as the team members call out discoveries or hunch over puzzles in groups. Most groups will leave the game with excitement and continue to discuss the activity online, also known as froth.

Nicholson distinguishes four basic game formats depending on the presence of a specific theme and narrative, and whether the puzzles are stand-alone or integrated into the narrative. In the more elaborate games, the puzzles are part of the storytelling. The most immersive experience may come from games designed with attention to player motivations, physical environment, intellectual challenges, and emotions [8].

The puzzle design is either path-based or sequential. In path-based designs, the team is presented with several different puzzles at the same time and each of the paths are needed to solve a meta-puzzle, which will unlock the next stage or the victory. The team can split into smaller groups to solve path-based puzzles. The sequential game design presents the players with one puzzle at a time that will unlock the next puzzle in sequence. The sequential design requires the entire team work together. The puzzle structure is often displayed in a flowchart.

The most frequent types of puzzles are searching hidden objects; using light; counting; noticing something obvious in the room; symbol substitution with a key; using something in an unusual way; searching images; assembly of an object.

Nearly all escape room facilities provide a gamemaster to ensure fair player experience. The gamemaster may monitor the players via video, help the players if stuck or frustrated, and ensure safety. Gamemasters may also give hints, either on request or at timed intervals.

Nicholson's survey indicates that the average player's success rate is 41%.

Escape rooms have become hugely popular, with over 2300 event facilities currently available in the USA alone, where only a dozen existed in 2014 [9]. Educational use of escape rooms is also increasing [10], and specific tutorials are available for implementation in higher education [11], including undergraduate medical teaching [12].

Curricular context
This serious game is implemented in the undergraduate medical curriculum in obstetrics and gynecology at the University of Oslo.

Prerequisite knowledge
The students are asked to prepare for the session by going through texts, videos, and images on the class webpage. The online material covers assisted reproduction, early pregnancy, ultrasound examination of the female pelvis and the testicles, IVF laboratory, etc. The students are also asked to review relevant textbook chapters (e.g., chapters 8 and 9 of Gynecology by Ten Teachers, 19th edition, Hodder Arnold, London.)

Educational context
The game is played during the clinical class in reproductive medicine for small groups (3 – 4 students).

The teaching begins with a clinical round, where the students spend 2 hours with one-by-one teaching with a gynecologist at the outpatient clinic (4 - 6 consultations). The most

common clinical issues at this clinic are infertility, assisted reproduction treatment, ovulation induction, early pregnancy control, and failed pregnancy. All students have the opportunity to observe or perform transvaginal ultrasound examination and acquire some overview of the pelvic anatomy as seen on the ultrasound scanner.

After the clinical round, the group reconvenes outside of the game room.

Implementation

1. Safety

The game is played in a clinical examination room using real clinical instruments. Safety of the players is the responsibility of the gamemaster.

Recommended safety checklist:

- Visit the room in advance and familiarize with all amenities, instruments, and storage cabinets.
- Secure items that can cause injury or get damaged if fallen or moved.
- Remove unnecessary clinical instruments, sharp or contaminated items, and safely lock in storage cabinets.
- Place and secure all props, including the lock box, to avoid falling object injuries.
- Clean and disinfect surfaces that might be contaminated during patient visits.
- Mark non-playable items, cabinets, and drawers with red dot stickers.
- Inspect the ultrasound scanner for damaged cables. Lock the wheels of the scanner.
- Clean and disinfect the transducer, the keyboard, and switches.
- Inform the students on professional conduct.
- Use CCTV, e.g. a baby monitor, to follow the gameplay.
- Be available in the vicinity of the room during the game.

2. Time requirements

Preparation of the room, equipment, and props, 15 min
Gameplay, 30 min
De-briefing, 30 min
Clean-up, 15 min
Total, 1h 30 min

3. Group size

For optimal game experience, the recommended group size is 3-5.

4. Location

Outpatient clinical room with sufficient space for the student group to move around. The group should be undisturbed during gameplay and de-briefing, appr. 60 min.

Amenities:

- desk,
- shelf or desk to place the locker box,
- gynecological examination chair.

5. Equipment, props

- «Letter from the doctor» in envelope
- Ultrasound scanner with transvaginal transducer
- Ultrasonography transmission gel and protective sheath for the transducer
- Intrauterine pregnancy endovaginal ultrasound training model (Blue Phantom, item No. BPOB1200, [external link])
- Privacy skirt to cover the model
- On the desk
  - Melloni's Illustrated Dictionary of Obstetrics and Gynecology, Parthenon Publishing, New York, 2000 [external link]
  - Daily desktop calendar, teared off to show 14-08-2020. [Remember to adjust the dates in the journal notes according to the year of your calendar.]
  - Three patient journals
- Locker box with digital combination lock, set to «20019049# [external link]
  - Address book with the name of the patients and solution to the "Phone number" puzzle. The solution is the phone number of the gamemaster. One or more digits of the number is concealed as the digit of CRL.
  - Pregnancy wheel with CRL scale
- Operating instructions for the locker box taped outside the box
- CCTV or baby call monitor [external link]
- Countdown clock with large display [external link]

6. Preparations

- Check the letter, journals and address book for notes or hints from previous groups. Print a clean copy if necessary.
- Close the door to the room to be undisturbed.
- Perform the Safety checklist.
- Clean the desk for all unnecessary items.
- Place the calendar, the dictionary and the journals on the desk.
- Place the phantom on the examination chair and cover with privacy skirt.
- Place the locker box.
- Open the locker box with code «20019049#». Place inside the address book and the pregnancy wheel. Close the locker box.
- Turn on the ultrasound scanner, set the transvaginal transducer. It is recommended to label the most essential buttons and switches with post-it stickers (freeze/unfreeze; caliper; zoom in/out).

- Place the transmission gel and the sheath cover around the instrument.
- Place the baby call so that you can view the group around the ultrasound scanner. Turn on the baby call, test audio and video.
- Place the countdown timer on the desk and set to 30 min.

7. Starting the game

- Greet the group outside the room.
- Read the following text to the group

Dear group,

Your task is to solve a situation in this room. The doctor who had been working here is now called to OR with short notice, and she asked me to give you this letter.

It is urgent, you have 30 minutes to complete her assignment.

Everything you need is on display in the room, you are not supposed to open drawers or cupboards, or use any item marked with a red dot.

At some point you may need to operate the ultrasound scanner. Please only use the keys and switches that are labelled.

You don't need any additional information that you cannot collect from the items you find on the desk, so do not waste time by internet browsing.

You must follow a professional conduct inside.

I will follow you with the Babycall but no recordings will be kept – if you need a hint, just ask, you can get 2 hints 10 min apart.

Are you ready?

- Give the "Letter from the doctor" to one of the students.
- The group enters the room.
- Turn on the countdown timer.

8. Gameplay

The gamemaster observes the game with the baby monitor, takes notes of mistakes or uncertainties that will be addressed during de-briefing. The gamemaster can provide hints if requested or if the group gets stuck in the game.

9. Ending the game

The group wins the game if they call the phone number of the gamemaster and give the right advise about follow-up (e.g., the patient is pregnant in week 8 and she should book appointment for regular antenatal care at week 12 in pregnancy). If the group fails to call the number within 30 min, the gamemaster should enter the room and end the game.

10. De-briefing

De-briefing is essential to achieve the learning goals of the class. It is recommended to commit 30 – 45 min to address the most common errors, uncertainties or gaps in knowledge. Many gaps are surprisingly non-obvious and might not be observed during gameplay.

Suggested questions and discussion points:

- Rehearse proper examination technique. (Ask a student who hasn't operated the scanner to follow your instructions.)
- Positioning the transducer, orientation on the screen.
- Pelvic anatomy, including the position of the vagina, bladder, vaginal fornices, vesicouterine and rectouterine pouch, etc.
- Ultrasound features of early pregnancy (localization, number of gestational sacs, heart beat (not on the phantom!), CRL),
- Why this mass is not a fibroadenoma, which is a common sidetrack during gameplay?
- Gestational age of pregnancies conceived spontaneously or with IVF.
- Determination of gestational age using the pregnancy wheel.
- Interpretation of the menstrual calendar.
- Ectopic pregnancy.
- Quality of teamwork.
- What did you learn from this experience?
- Did a leader emerge? How? Did this change at all during the experience?

11. Post-class activities

The gamemaster cleans and boxes up the phantom, cleans and disinfects the transducer, and tidies the desk. It is recommended to check the props for notes or hints and print a clean copy for the next game.

It is recommended to keep a reflection note after each session and review the notes regularly to improve the game.

11. Evaluation

It is recommended to invite feedback from students. Several online platforms provide an easy way to create feedback forms (e.g. SurveyMonkey, Doodle, etc).

Suggested items for feedback form:

Item	Remark
What were you satisfied with?	Open text, max 50 words
What should be improved?	Open text, max 50 words
Overall score.	Scale A – E
Will you rate the teamwork during the Escape Room? (11 questions)	Yes/No
There appeared to be a team leader who coordinated the discussion	4-level Likert scale
The team leader facilitated the discussion rather than dominated it	4-level Likert scale
Members of the team came prepared to the class	4-level Likert scale
All members of the team contributed appropriately	4-level Likert scale
Team members had respect, confidence and trust in one another	4-level Likert scale
Team members listened and payed attention to each other	4-level Likert scale
Team members listened to and considered the input of others before pressing their own ideas	4-level Likert scale
The opinions of the team members were valued by other members	4-level Likert scale
Team members appeared to feel free to disagree openly with each other's ideas	4-level Likert scale
Team members sought out opportunities to work with others on problem solving	4-level Likert scale
Team interactions were friendly	4-level Likert scale
One new thing that you learned today	Open text, max 50 words
One new thing, either positive or negative, that you observed today about teamwork	Open text, max 50 words

Items 5–17 are adapted from Lyons et al. [13].

References

1. Robson, K., et al., *Is it all a game? Understanding the principles of gamification.* Business Horizons, 2015. **58**(4): p. 411-420.
2. Boeker, M., et al., *Game-based e-learning is more effective than a conventional instructional method: a randomized controlled trial with third-year medical students.* PLoS One, 2013. **8**(12): p. e82328.
3. Cardozo, L.T., et al., *Effect of a puzzle on the process of students' learning about cardiac physiology.* Adv Physiol Educ, 2016. **40**(3): p. 425-31.
4. Patel, J., *Using Game Format in Small Group Classes for Pharmacotherapeutics Case Studies.* American Journal of Pharmaceutical Education, 2008. **72**(1): p. 21.
5. Gorbanev, I., et al., *A systematic review of serious games in medical education: quality of evidence and pedagogical strategy.* Medical Education Online, 2018. **23**(1): p. 1438718.
6. Maheu-Cadotte, M.A., et al., *Effectiveness of serious games and impact of design elements on engagement and educational outcomes in healthcare professionals and students: a systematic review and meta-analysis protocol.* BMJ Open, 2018. **8**(3): p. e019871.
7. Nicholson, S., *The State of Escape: Escape Room Design and Facilities*, in *Meaningful Play 2016*. 2016: Lansing, Michigan.
8. Heikkinen, O. and J. Shumeyko, *Designing an escape room with the Experience Pyramid model*. 2016, Haaga-Helia University of Applied Sciences: Helsinki, Finland.
9. *July 2018 Escape Room Industry Growth Study*. 2018 [cited 2018 21.10.2018]; Available from: https://roomescapeartist.com/2018/07/28/2018-escape-room-industry/.
10. Stone, Z., *The rise of educational escape rooms.* The Atlantic, 2016.
11. Clarke, S., et al., *EscapED: A Framework for Creating Educational Escape Rooms and Interactive Games to For Higher/Further Education*. Vol. 4. 2017.
12. Friedrich, C.L., et al., *Healthcare escape room design guidebooks.* , in *Technology #20180272-20180273*. 2018, University of Minnesota.
13. Lyons, K.J., et al., *Jefferson Teamwork Observation Guide (JTOG): An Instrument to Observe Teamwork Behaviors.* J Allied Health, 2016. **45**(1): p. 49-53.

Appendix

A. Flowchart

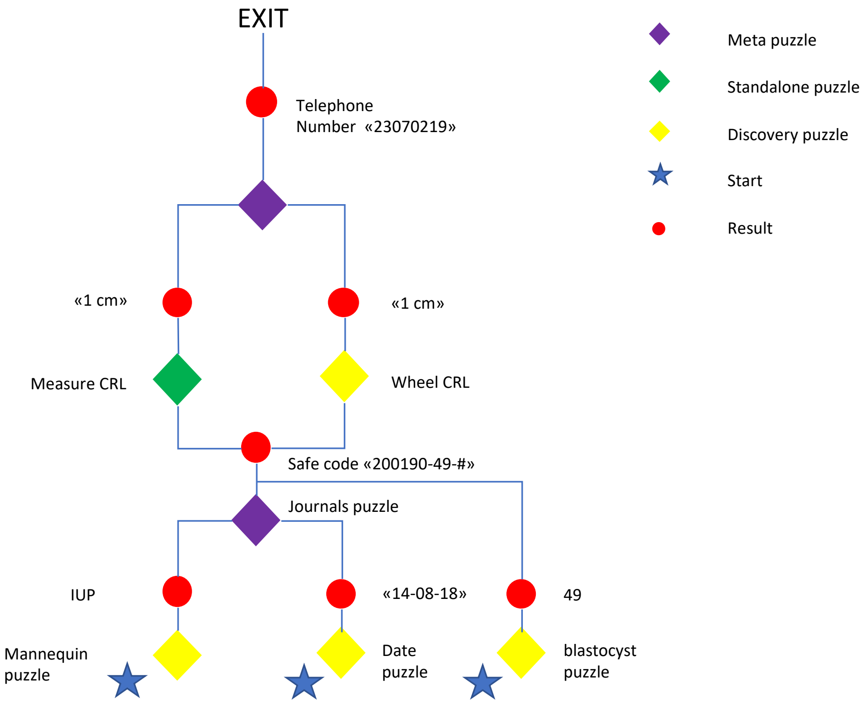

B. Description of the puzzles

Date puzzle

Description: The desktop calendar shows 14-AUG-2018, the date is needed to interpret the journal notes, pregnancy dates, etc.
Requires: Finding the desktop calendar
Required by: Journals puzzle, Wheel CRL puzzle
Difficulty: Low
Result: 14-AUG-2018

Blastocyst puzzle

Description: Part of the locker box code is the page number for the term ‘blastocyst’ in the medical dictionary.
Requires: Recognizing blastocyst on the image; finding the medical dictionary
Required by: Safe code puzzle
Difficulty: Medium
Result: 49

IUP puzzle

Description: The mannequin displays an intrauterine pregnancy (sonolucent ring in the uterine cavity that is asymmetrically located to the endometrium with a distinct embryo of appr 11 mm).
Requires: Finding the mannequin and operating the ultrasound scanner
Required by: Journals puzzle, Measure CRL puzzle
Difficulty: High
Result: IUP

Journals puzzle

Description: The 3 patient journals describe 3 very distinct clinical situations, whereas just one case matches the IUP puzzle.
Requires: IUP puzzle, Date puzzle
Required by: Safe code
Difficulty: High
Result: 200190

Safe code

Result: 20019049#

Measure CRL puzzle

Description: The telephone number requires the team to obtain the CRL. The CRL can be obtained by measuring directly on the mannequin with ultrasound.
Requires: Safe code, IUP puzzle
Required by: Telephone number
Difficulty: Medium
Result: 11 mm

Wheel CRL puzzle

Description: The telephone number requires the team to obtain the CRL. The CRL can be obtained by reading from the gestation wheel, using the date on the desktop calendar and the last menstrual period or the IVF treatment date from the journal.
Requires: Safe code, finding the gestation wheel, Date puzzle
Required by: Telephone number
Difficulty: High
Result: 11 mm

Telephone number
Result: 23070219

C. Letter from the doctor

14-08-2018

Dear all,

I had an awful morning this Tuesday!

After my night shift, I stepped in the outpatient clinic and now I must hurry to the OR, while the paperwork is still incomplete.

I have seen three patients today.

You will find all the three journals on the desk.

I need you to call my last patient and instruct her about follow-up.

The address book with the phone number is in the locker. You will find everything to open the locker on the desk.

Good luck!

Hint: ultrasound findings of the mannequin will exactly match what I have seen on the last patient. Can you find her journal?

Safe code:

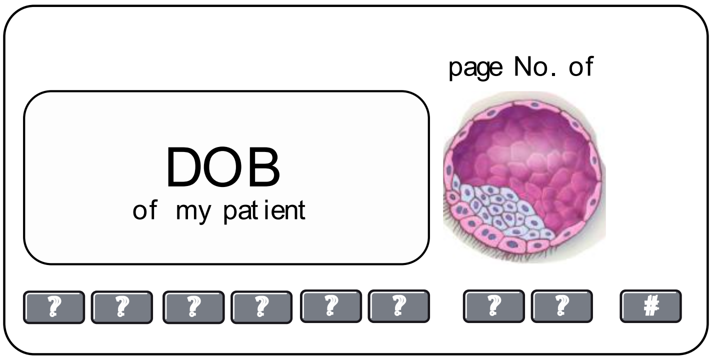

D. Journals

Oslo University Hospital	Patient ID<br>Annod, Elvira DOB 20-01-1990	**Treatment record**<br>**Department of Reproductive Medicine**

Date		Clinical data		Protocol	Treatment type
Last menstruation	20-06-2018	Diag. male factor	AMH 34 pmol/l	☐ long downregulation<br>☒ GnRH antagonist<br>☐ ovulation induction<br>☐ other	intracyto-plasmic sperm injection
GnRH agonist start	not given	Age 28 yrs			
FSH start	22-06-2018	BMI 24,6			

Stimulation																
Date	22/6					27/6			30/6			3/7		5/7		7/7
Day	1	2	3	4	5	6	7	8	9	10	11	12	13	14	15	16
recFSH (112IU/d)	x	x	x	x	x	x	x	x	x	x	x					
GnRH antagonist						x	x	x	x	x	x	x				
hCG (6500 IU)												20:30				
Oocyte collection														08:30		
Embryo transfer																14:00
R follicle (mm) 1						<10			13			19				
2									13			18				
3									11			15				
4									<10			11				
5																
6																
L follicle (mm) 1						<10			13			18				
2									12			16				
3									<10			12				
4																
5																
6																
Se estradiol (nM)						0,2			0,9			6,2				
Endometrium(mm)									6,3			10,1				
Signature																
Notes																

Oocyte collection						
Date<br>05-07-18	Time<br>08:30	No. of oocytes<br>10	No. injected<br>8	No.fertilized<br>6	MD/sign	Lab/sign
Notes<br>Collected 10 oocytes, 8 mature used for microinjection, 6 fertilized with 2 pronuclei.					☐ ID controlled<br>☐ Consent signed	

Embryo transfer							
Date<br>07-07-18	Time<br>14:00		Embryo_quality<br>4 cell, type 1.0		No.frozen<br>2	MD/sign	Lab/sign
Notes<br>Transferred one top quality embryo on day 2. Two embryos frozen.						☐ ID controlled<br>☐ Consent controlled	

Intrauterine insemination					
Date	Time	Sperm native	Sperm prepared	MD/sign	Lab/sign
Notes				☐ ID controlled<br>☐ Consent controlled	

Oslo University Hospital	Patient ID<br>Annod, Elvira, DOB 20-01-90<br>Hammarsborggate 13, 0110 Oslo	Medical record<br>**Department of Reproductive Medicine**

Outpatient note

Date 02-03-2018

Referral diagnosis:
N97 primary infertility

Duration of infertility: 2 years

Family/social:
Married couple. Elvira is a lawyer and partner in a law firm. Her husband Juhani is a key accountant manager in a consultancy firm.

Medical history: Mostly healthy.
Allergies: pollen
Medication: Cetrizin 10 mg x 1
Stimulants: non-smoker.

Medical exam:
Height 165 cm, weight 67 kg.

Gynecological history: gravida 0, para 0.
Menarche at 12 years of age. Regular menstrual cycles 5-7/28, rarely over 30 days. Mild dysmenorrhea on the first menstrual day.
Cervical screening: normal
Serum anti-Müllerian hormone (AMH) 34 pmol/L (ref. range 4 – 84 pmol/L).

Gynecological exam:
Vulva, vagina, portio normal.
Transvaginal ultrasound: anteverted uterus, irregular endometrium with a possible small polyp of 12 mm. Ovaries with multiple small antral follicles, more than 15 on each side.

Partner:
Annod, Juhani, DOB 11-05-85
Medical history: negative
Allergies: none
Medications: none
Reproduction: no previous pregnancies.
Sperm test: reduced sperm concentration (7 millions/mL, ref. > 10), reduced motility (20%, ref > 40%).

Diagnosis:
Primary infertility due to male factor.

Plan:
Assisted reproduction treatment with intracytoplasmic sperm injection. Ovarian stimulation with recombinant FSH.
Information about the treatment, costs, complications, etc.
Treatment is scheduled for July 2018.

Ambulatory note

Date 14-08-2018

Reason for appointment: Pregnant after assisted reproduction treatment.

07-07-2018: Transfer of a single top-quality embryo.
19-07-2018: Serum hCG 40 IU/L (ref. range for pregnancy > 20 IU/L)

Transvaginal ultrasound scan:
–– TO BE COMPLETED ––

Oslo University Hospital

Menstrual Record Chart

Department of Reproductive Medicine

Patient: Donna, Anna

Address: Oslo

Year: 2018

Month	1	2	3	4	5	6	7	8	9	10	11	12	13	14	15	16	17	18	19	20	21	22	23	24	25	26	27	28	29	30	31	Number of days from start of period to beginning of next
January															x	x	x	•	•	•	•								I	I	I	
February												x	x	x	•	•	•	•								I	I	I				
March												x	x	x	•	•	•	•								I	I	I				
April									x	x	x	•	•	•	•								I	I	I							
May							x	x	x	•	•	•	•							I	I	I										
June		•	•															I	I	I												
July																																
August																																
September																																
October																																
November																																
December																																

Don't forget to have this chart with you when you call or visit your doctor

TYPE OF FLOW [x] Normal [+] Exceptionally light ■ Exceptionally heavy [•] Spotting I - intercourse

web0709

Outpatient note

Date 10-12-2017

Referral diagnosis:
N97 primary infertility. The couple reports inability to conceive since January 2017.

Family/social:
Married couple. Anna is a PhD student at the UiO. Johannes is an IT consultant.

Medical history:
Reports asthma and allergy (pollen, kiwi and pineapple). Otherwise healthy.

Medications:
Nasal fluticasone (Avamys) and Loratidin when needed.

Stimulants: Non-smoker. Teetotal.

Gynecological history:
Gravida 0, para 0.
Menarche 11-12 years. Some dysmenorrhea during adolescence. She has taken OCP from 18 years of age and stopped after she was married in December 2017. Regular menstrual periods 7/28-32 days with moderate dysmenorrhea. Laparoscopy in June 2017 excluded peritoneal endometriosis and found patent Fallopian tubes.
No history of sexually transmitted disease. Regular normal cervical cytology exams.
Serum anti-Müllerian hormone (AMH) 20 pmol (ref. range 4 – 84 pmol/L).

Medical exam:
Height 165 cm, weight 60 kg.

Gynecological exam:
Vulva, vagina, portio normal.
Transvaginal ultrasound scan:
Retroverted uterus with regular endometrium 10 mm in proliferative phase. The right ovary contained 4 antral follicles and the left ovary contained 5 antral follicles and 1 dominant follicle.

Partner:
Don, Johannes, DOB 10-03-1981

Medical history: negative
Allergies: none
Medications: none
Reproduction: no history of STI, no previous pregnancy
Semen analysis: normal concentration and motility.

Diagnosis: Unexplained infertility

Treatment plan: Given the relatively short history, spontaneous conception in timed intercourse is advised. Use of a menstrual calendar is recommended.
Control after 6 months.

Outpatient note

Date 14-08-2018

Reason for appointment:
Follow-up for primary infertility.

The couple had been advised to attempt spontaneous conception in timed intercourse. She reports morning sickness and breast tenderness. Took positive home test for urinary hCG 02-06-2018. Coming now for review of menstrual chart and ultrasound scan.

Transvaginal ultrasound scan:
–– TO BE COMPLETED ––

Oslo University Hospital	Patient ID<br>Lammermoor, Lucia DOB 01-06-1984<br>Skottlandsgaten 16, 0440 Oslo	Medical record<br>**Department of Reproductive Medicine**

Outpatient note

Date 14-08-2018

Reason for referral:
Abdominal pain in early pregnancy

Family/social: health care worker in permanent job

Medical history: Mostly healthy
Allergies: none
Medications: none
Stimulants: non-smoker, 1-2 units of alcohol a week

Gynecological history:
Gravida 3, para 1. Termination of pregnancy in 2002. Normal vaginal term delivery in 2007. Right salpingectomy for ectopic pregnancy in 2017.

Current complaint:
Sudden onset of left-sided abdominal pain this morning. Light vaginal spotting started yesterday.
She has recently undergone IVF-treatment with oocyte collection 15-07-2018 and intrauterine transfer of a single top-quality embryo 17-07-2018. Home pregnancy test was positive 3 weeks after embryo transfer.

Status presens:
BP 111
Pulse 75
Temp 37
Respiration 16
O2 saturation 100
Height 163
Weight 54
Cor normal
Pulmones normal

Vaginal inspection: light brown discharge from portio.
Bimanual palpation: left adnexal tenderness

Blood work:
31-07-2018 – serum hCG 6.6 IU/L
12-08-2018 – serum hCG 2757 IU/L
14-08-2018 – serum hCG 3010 IU/L
14-08-2018 – hemoglobin 12 g/dL (11.7 – 15.3)
14-08-2018 – hematocrit 0.38 (0.35 – 0.46)
14-08-2018 – Na 139 mM (137 - 145)
14-08-2018 – K 4.0 mM (3.6 – 4.6)
14-08-2018 – Kreatinin 58 µM (45 - 90)
14-08-2018 – Albumin 44 g/L (36 - 48)

Transvaginal ultrasound scan:
Anteverted uterus, endometrial thickness 7 mm, empty uterine cavity. Right ovary with corpus luteum cyst. Left adnexa with dilated tube 16 x 20 mm, free fluid in cul-de-sac 11 mm.

Conclusions, plan:
— TO BE COMPLETED —

E. Address book page

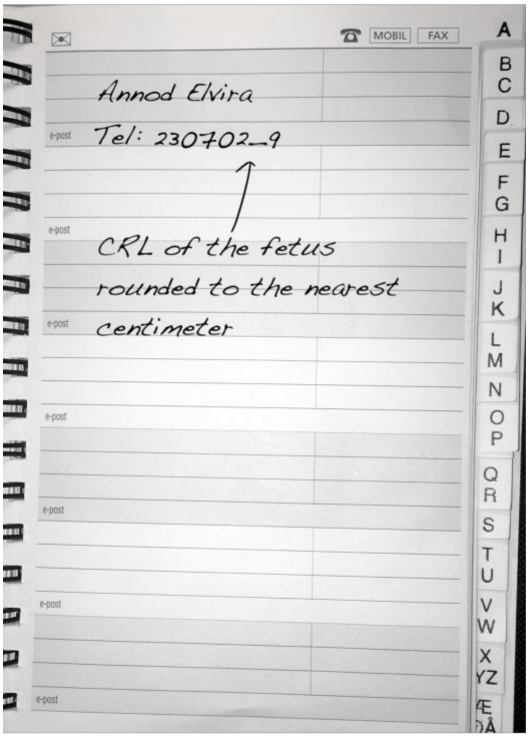

Supplement: Supplementary file 1 — Supplementary Material 1 [file 12909_2024_6352_MOESM1_ESM.pdf]
