# Supplementary material for: Moderate benefit of escape room game on learning outcome in medicine
Source: BMC Med Educ. 2024 Nov 23;24:1353. doi: 10.1186/s12909-024-06352-8 (PMC11585938; doi:10.1186/s12909-024-06352-8)
Supplement: Supplementary file 2 — Supplementary Material 2 [file 12909_2024_6352_MOESM2_ESM.docx]

## Student evaluation form

| **Item** | **Remark** |
| --- | --- |
| What were you satisfied with? | Open text, max 50 words |
| What should be improved? | Open text, max 50 words |
| Overall score. | Scale A – E |
| Will you rate the teamwork during the Escape Room? (11 questions) | Yes/No |
| There appeared to be a team leader who coordinated the discussion | 4-level Likert scale |
| The team leader facilitated the discussion rather than dominated it | 4-level Likert scale |
| Members of the team came prepared to the class | 4-level Likert scale |
| All members of the team contributed appropriately | 4-level Likert scale |
| Team members had respect, confidence and trust in one another | 4-level Likert scale |
| Team members listened and paid attention to each other | 4-level Likert scale |
| Team members listened to and considered the input of others before pressing their own ideas | 4-level Likert scale |
| The opinions of the team members were valued by other members | 4-level Likert scale |
| Team members appeared to feel free to disagree openly with each other's ideas | 4-level Likert scale |
| Team members sought out opportunities to work with others on problem solving | 4-level Likert scale |
| Team interactions were friendly | 4-level Likert scale |
| One new thing that you learned today | Open text, max 50 words |
| One new thing, either positive or negative, that you observed today about teamwork | Open text, max 50 words |

Items 5–17 are adapted from Lyons KJ, Giordano C, Speakman E, Smith K, Horowitz JA: **Jefferson Teamwork Observation Guide (JTOG): An Instrument to Observe Teamwork Behaviors**. *J Allied Health* 2016, **45**(1):49-53.
